# Insight into the Mechanism of Intramolecular Inhibition of the Catalytic Activity of Sirtuin 2 (SIRT2)

**DOI:** 10.1371/journal.pone.0139095

**Published:** 2015-09-25

**Authors:** Jinyu Li, Franziska Flick, Patricia Verheugd, Paolo Carloni, Bernhard Lüscher, Giulia Rossetti

**Affiliations:** 1 Computational Biomedicine, Institute for Advanced Simulation IAS-5 and Institute of Neuroscience and Medicine INM-9, Forschungszentrum Jülich, 52425, Jülich, Germany; 2 Institute of Biochemistry and Molecular Biology, RWTH Aachen University, 52057, Aachen, Germany; 3 Computational Biophysics, German Research School for Simulation Sciences, Forschungszentrum Jülich, 52425, Jülich, Germany; 4 Jülich Supercomputing Centre, Forschungszentrum Jülich, 52425, Jülich, Germany; 5 Department of Oncology, Hematology and Stem Cell Transplantation, RWTH Aachen University, Aachen, Germany; University of Edinburgh, UNITED KINGDOM

## Abstract

Sirtuin 2 (SIRT2) is a NAD^+^-dependent deacetylase that has been associated with neurodegeneration and cancer. SIRT2 is composed of a central catalytic domain, the structure of which has been solved, and N- and C-terminal extensions that are thought to control SIRT2 function. However structural information of these N- and C-terminal regions is missing. Here, we provide the first full-length molecular models of SIRT2 in the absence and presence of NAD^+^. We also predict the structural alterations associated with phosphorylation of SIRT2 at S331, a modification that inhibits catalytic activity. Bioinformatics tools and molecular dynamics simulations, complemented by *in vitro* deacetylation assays, provide a consistent picture based on which the C-terminal region of SIRT2 is suggested to function as an autoinhibitory region. This has the capacity to partially occlude the NAD^+^ binding pocket or stabilize the NAD^+^ in a non-productive state. Furthermore, our simulations suggest that the phosphorylation at S331 causes large conformational changes in the C-terminal region that enhance the autoinhibitory activity, consistent with our previous findings that phosphorylation of S331 by cyclin-dependent kinases inhibits SIRT2 catalytic activity. The molecular insight into the role of the C-terminal region in controlling SIRT2 function described in this study may be useful for future design of selective inhibitors targeting SIRT2 for therapeutic applications.

## Introduction

**Fig 1:**
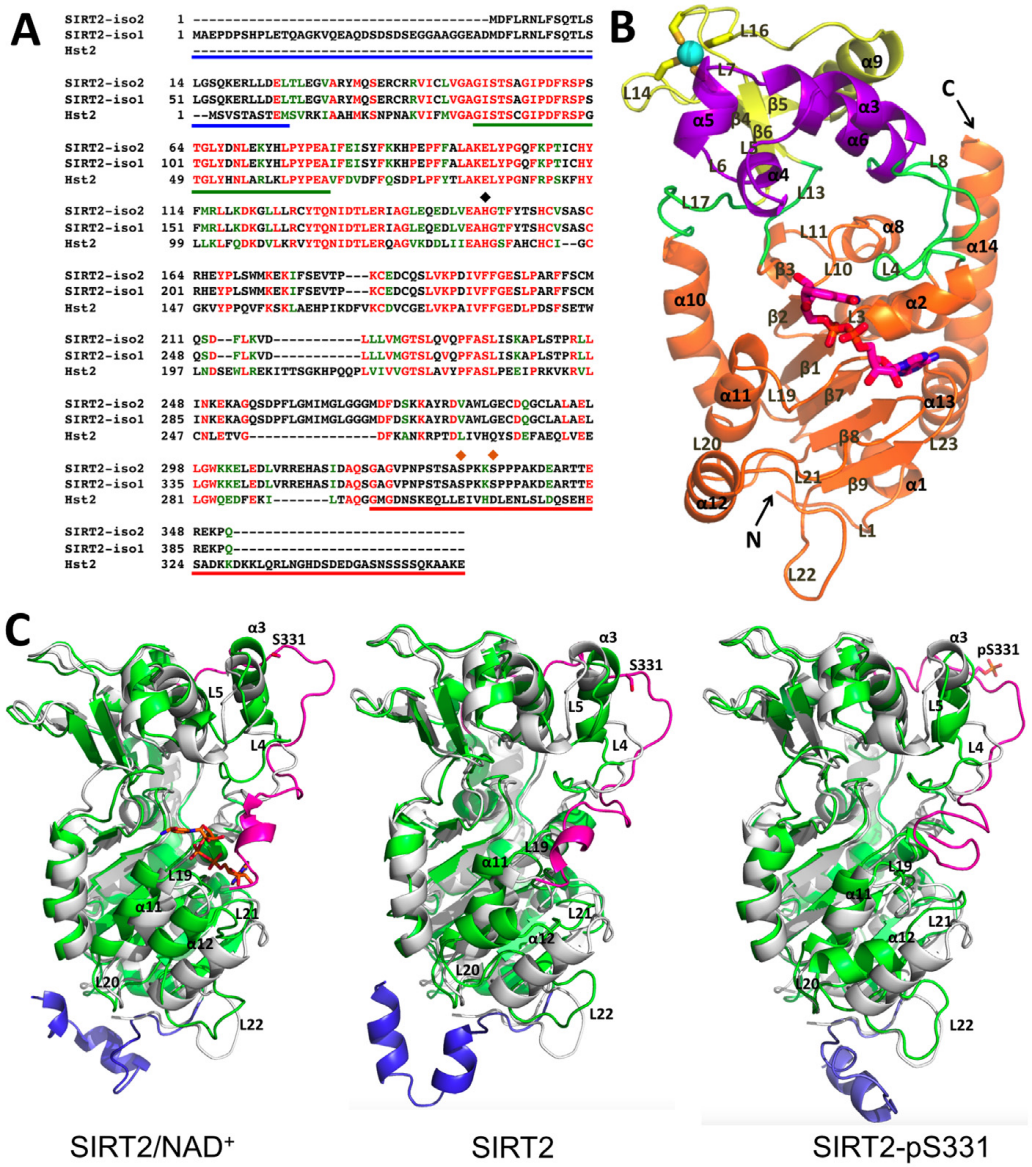
Structural features of SIRT2. **(A)** Primary sequences of SIRT2 isoform 1 (SIRT2-iso1) and isoform 2 (SIRT2-iso2) aligned with that of Hst2 from *S*. *cerevisiae* (obtained using the BLAST webserver (http://blast.ncbi.nlm.nih.gov/Blast.cgi)). Identical and structurally similar residues are indicated in red and green, respectively. Solid lines indicate the N-terminal (NT, blue) and C-terminal (CT, red) regions, and so-called NAD^+^ cofactor-binding loop (green). The catalytic center residue H150 (black diamond) and the phosphorylation sites S331 and S335 (orange diamonds) are shown. **(B)** X-ray structure of the catalytic core (CC) domain of human SIRT2 (PDB ID: 1J8F [13]). The CC consists of (i) a Rossmann fold (orange), made of six β-strands forming a parallel β–sheet and six α-helices, (ii) a small domain made up by a zinc binding pocket and a hydrophobic pocket, which contains a three-stranded antiparallel β-sheet (yellow), an α-helix (yellow) and a zinc ion (light blue) coordinated by four cysteine residues (C195, C200, C221 and C224 (yellow sticks)), and four α-helices forming a hydrophobic pocket (purple), and (iii) four connecting loops (green). NAD^+^ is absent in the crystal structure. It has been included here by superposing the CC of SIRT2 with that of Sir2-Af1 [14], for which a structure with NAD^+^ is available (NAD^+^ is in magenta). N and C indicate the N-terminal and C-terminal ends of the CC, respectively. **(C)** Structural comparison of the CC domain obtained from X-ray structure determinations and of the modeled SIRT2/NAD^+^ complex, the modeled SIRT2 and the modeled SIRT2-pS331. The CC crystal structure and our models are colored in gray and green, respectively. The NT and CT are colored in blue and pink, respectively. The NAD^+^ cofactor in the CC of the modeled SIRT2/NAD^+^ is represented by orange sticks. The S331/pS331 residues in the CT are represented by pink sticks.

The human NAD^+^-dependent enzyme Sir2-like protein 2 (SIRT2 hereafter, Fig 1) has been associated with several age-related diseases, including diabetes, cardiovascular diseases, neurodegenerative disorders and cancer [1–7]. Hence, targeting SIRT2 may be of therapeutic relevance. SIRT2 is expressed as two functionally similar isoforms (isoform 1 and 2). Isoform 2 (352 amino acids) lacks the first 37 N-terminal amino acids compared to isoform 1. Both isoforms have the same in vitro and in vivo deacetylation activity [8], i.e. SIRT2 deacetylates lysine residues in a variety of target proteins, thus antagonizing acetyltransferases [9,10]. In the current study, we focus on isoform 2 of SIRT2, which is the isoform highly expressed in the myelin-enriched fractions of adult mouse brains and in the cytoplasm of murine cerebellar granule cells [1,2]. Inhibitors of the catalytic activity of SIRT2 have been shown to interfere with α-synuclein toxicity [4], which is associated with neurodegeneration in Parkinson’s disease, and with tumorigenesis [7]. However these inhibitors are non-selective as they bind also to other members of the sirtuin family [4,11,12].

Previously, we found that cyclin-dependent kinase 2 (CDK2) and CDK5 can phosphorylate SIRT2 at serine (S) 331 of isoform 2 (corresponds to S368 in isoform 1). This serine residue is located within the naturally disordered C-terminal region (CT, residues 320–352 in isoform 2). This phosphorylation inhibits the enzymatic activity of SIRT2 both *in vitro* and in cells [15]. Gain- and loss-of-function studies revealed that SIRT2 interferes with neurite outgrowth in postmitotic hippocampal neurons, which is antagonized by CDK-dependent phosphorylation [15]. In agreement with our findings defining S331 as phosphorylation site, the corresponding residue S368 in SIRT2 isoform 1 was identified as a target of CDK1 [8]. SIRT2 overexpression delays cell proliferation, which is dependent on S368 phosphorylation [8]. Together these findings suggest that phosphorylation of the CT affects SIRT2 activity and hence this naturally disordered region might function as a regulatory domain. In an effort to evaluate whether this is indeed the case, we have used bioinformatics tools and molecular dynamics (MD) simulations to provide molecular models of the full-length protein, with and without the NAD^+^ cofactor (SIRT2 and SIRT2/NAD^+^, respectively, hereafter), as well as with a phosphorylated S331 without NAD^+^ (SIRT2-pS331 hereafter). The predictions are based on the structured catalytic core (CC, residues 26–319 in isoform 2) deduced from the crystal structure [13]. The calculations are complemented by enzymatic essays. Our *in silico* and *in vitro* approaches provide a consistent picture based on which we suggest that the CT functions as an autoinhibitory region by partially occluding the NAD^+^ binding site in SIRT2 or stabilizing the NAD^+^ in a non-productive state in SIRT2/NAD^+^. This occlusion is increased by phosphorylation at S331.

## Materials and Methods

### Computational chemistry

#### Modeling of SIRT2/NAD^+^

**Fig 2:**
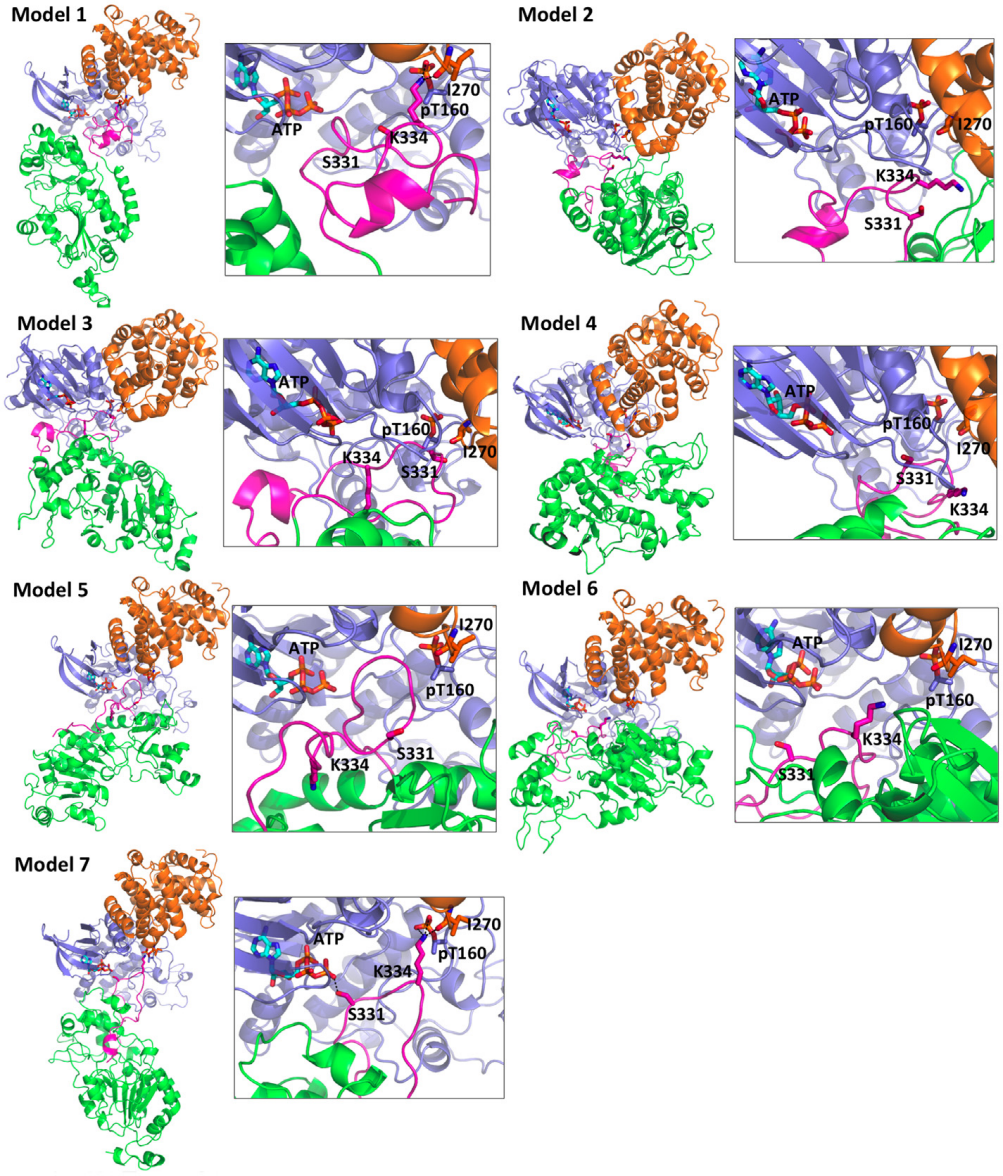
Structural models of the lowest interaction energy docking structure of SIRT2 in complex with cCDK2 in the largest cluster. The structures of Models 1–6 were initially generated by homology modeling, while Model 7 was generated by *ab initio* modeling (see Materials and Methods for details). The cartoon representations of CDK2, cyclin A, and SIRT2 are colored in blue, orange, and green, respectively. The CT of SIRT2 is colored in pink.

SIRT2 contains the CC domain (Fig 1B), for which structural information is available (PDB ID: 1J8F [13]) and two unstructured regions, the N-terminal (NT) region and the CT. The CC structure includes a zinc ion, coordinated by four cysteine residues (C195, C200, C221 and C224) (Fig 1B), but lacks the NAD^+^ cofactor [13], which is present in the CC and necessary for the catalytic activity of sirtuins [14,16–19]. The CC shares the same domain architecture [13] and 26% sequence identity with Sir2-Af1 from *A*. *fulgidus* (PDB ID: 1ICI [14]). Hence, we have obtained an educated guess of the location of the NAD^+^ cofactor by superimposing the two structures. Next, we constructed structural models of the NT and CT regions by (i) homology modeling and (ii) *ab initio* modeling. (i) Because the BLAST webserver (http://blast.ncbi.nlm.nih.gov/Blast.cgi) was not able to find suitable templates for NT and CT, we split these regions into fragments, as described previously [20]. We identified 26 templates, 13 for NT and 13 for CT, from the BLAST webserver with sequence identities with the targets ranging from 48% to 100% (Table 1). 200 models of the full-length SIRT2 isoform 2 were then generated using the MODELLER 9v9 package [21]. (ii) *Ab initio* modeling [22], using the Robetta webserver [23], resulted in five full-length SIRT2 models. 172 models out of 200 generated from homology modeling and all 5 models from *ab initio* modeling turned out to have 90% or more residues in the most favored regions of Ramachandran plots [24]. For this analysis, the Procheck program [25] was used. Six different starting models (Models 1–6 in Fig 2), representing 86% of the 200 homology models, were identified by a cluster analysis [26]. One *ab initio* model (Model 7 in Fig 2) was selected among the five obtained. This shows the lowest difference (in terms of secondary structure and root-mean-square deviations (RMSD)) compared with the SIRT2 CC crystal structure [13].

**Table 1:** Templates of the N-terminal (NT) and C-terminal (CT) regions for SIRT2 homology models.

	Segment ID	PDB ID	Template sequence	Target sequence	Identity (%)	Ref.
**NT**	1	2CX6	D16-D36	D2–D23	55	[27]
	2	2OVJ	D424-L434	D2–D23	48	[28]
	3	3PSE	M1–L7	M1–L5	86	[29]
	4	2FCG	D26-L31	D2–L7	100	[30]
	5	2HEK	F295-E304	F8–D23	50	[31]
	6	3PCO	R195-T201	R5–T11	57	[32]
	7	1VE2	K169-E174	K18-D23	70	[33]
	8	2WSP	Q337-L342	Q17-L22	100	[34]
	9	3DJA	L179-G190	L4–R20	58	[35]
	10	1U7J	M1–R5	M1–R5	80	[36]
	11	1PWX	L211-E216	L14-E19	83	[37]
	12	3FMC	T239-R347	T11-R20	80	[38]
	13	1G0D	G445-R450	G15-R20	100	[39]
**CT**	14	3HJT	Q60-I65	Q318-V323	83	[40]
	15	1V8H	P65-P73	P324-P332	78	[41]
	16	2DFX	P58-P63	P332-P337	83	[42]
	17	1XG2	P105-A110	P338-A343	83	[43]
	18	1U3O	R58-Q66	R344-Q352	78	[44]
	19	2CUI	G44-T50	G322-T328	86	[45]
	20	1YPZ	K119-K125	K334-K340	86	[46]
	21	2X1L	G373-P379	G320-P326	71	[47]
	22	1YU3	S216-S222	S329-S335	100	[48]
	23	1YQ2	S789-A800	S319-A330	67	[49]
	24	3RQR	A2913-Q2924	A339-Q352	57	[50]
	25	1WQL	Q412-N419	Q318-N325	88	[51]
	26	1J3L	A113-K120	A330-K340	64	[52]

The PDB ID, template sequence, target sequence, template-target identity and reference are given.

#### MD simulations of SIRT2/NAD^+^

Each of the seven selected full-length SIRT2/NAD^+^ models, obtained from homology and *ab initio* modeling was inserted into a cuboid with edge lengths of 91 Å, 112 Å and 98 Å containing ~31,000 water molecules and six Na^+^ ions. We have determined the protonation states of the seven histidines in SIRT2 (H74, H90, H112, H150, H157, H165 and H312) by combining visual inspection for predicting possible interactions (hydrogen bond patterns or salt bridges) and by using two servers (WHAT IF [53] and H++ [54]) evaluating the local effects of the flanking amino acids on histidines’ p*K*_a_. We have additionally run short MD simulations (10 ns for each protonation state, with the same computational setup described below) on one of the seven models to evaluate the effect of the different protonation states on the SIRT2 structure. We have chosen the configuration with the lowest RMSD (Table 2) in respect to the SIRT2 crystal structure for following productive MD simulations, i.e. H74 (Nε), H90 (Nε), H112 (Nδ), H150 (Nε), H157 (Nε), H165 (Nε and Nδ) and H312 (Nδ)).

**Table 2:** Average RMSD values of backbone atoms of the CC of SIRT2 in a total of 240 ns MD simulations by using various histidine protonation states, relative to those in the X-ray structure.

**Histidine**	**Protonation state**							
	**a**	**b**	**c**	**d**	**e**	**f**	**g**	**h**
H74	HIE	HIE	HIE	HIE	HIE	HIE	HIE	HIE
H90	HIE	HIE	HIE	HIE	HIE	HIE	HIE	HIE
H112	HIP	HIP	HIP	HIP	HIP	HIP	HID	HID
H150	HID	HID	HIE	HIE	HIP	HIP	HID	HID
H157	HIP	HIE	HIP	HIE	HIP	HIE	HIP	HIE
H165	HIP	HIP	HIP	HIP	HIP	HIP	HIP	HIP
H312	HID	HID	HID	HID	HID	HID	HID	HID
RMSD (Å)	2.4±0.3	2.3±0.3	3.0±0.8	2.1±0.3	2.3±0.3	2.2±0.3	2.6±0.2	2.7±0.5
	**i**	**j**	**k**	**l**	**m**	**n**	**o**	**p**
H74	HIE	HIE	HIE	HIE	HIE	HIE	HIE	HIE
H90	HIE	HIE	HIE	HIE	HIE	HIE	HIE	HIE
H112	HID	HID	HID	HID	HIP	HIP	HIP	HIP
H150	HIE	HIE	HIP	HIP	HID	HID	HIE	HIE
H157	HIP	HIE	HIP	HIE	HIP	HIE	HIP	HIE
H165	HIP	HIP	HIP	HIP	HIP	HIP	HIP	HIP
H312	HID	HID	HID	HID	HIP	HIP	HIP	HIP
RMSD (Å)	2.9±0.4	2.0±0.4	2.4±0.3	2.2±0.4	2.3±0.3	2.4±0.4	2.1±0.3	2.4±0.4
	**q**	**r**	**s**	**t**	**u**	**v**	**w**	**x**
H74	HIE	HIE	HIE	HIE	HIE	HIE	HIE	HIE
H90	HIE	HIE	HIE	HIE	HIE	HIE	HIE	HIE
H112	HIP	HIP	HID	HID	HID	HID	HID	HID
H150	HIP	HIP	HID	HID	HIE	HIE	HIP	HIP
H157	HIP	HIE	HIP	HIE	HIP	HIE	HIP	HIE
H165	HIP	HIP	HIP	HIP	HIP	HIP	HIP	HIP
H312	HIP	HIP	HIP	HIP	HIP	HIP	HIP	HIP
RMSD (Å)	2.7±0.4	2.2±0.3	2.7±0.6	2.6±0.4	2.2±0.4	2.1±0.3	2.6±0.4	2.2±0.4

The three possible protonation states of histidine are represented by HID (Nδ nitrogen atom protonated), HIE (Nε nitrogen atom protonated) and HIP (both Nδ and Nε nitrogen atoms protonated). The possible combinations (a-x) are 24. Standard deviations (SD) of RMSD are also reported. The chosen configuration for MD simulations is state j with the lowest RMSD value compared with others.

Overall, the systems were neutral and underwent MD simulations using the GROMACS 4.5.5 code [55]. The all-atom AMBER ff99SB-ILDN force field [56–59] was used for the protein, Na^+^ and NAD^+^. The bonded plus electrostatic model and corresponding zinc AMBER force field (ZAFF) parameters (tetrahedral four cysteine zinc-bonding configuration [60]) were used for the zinc and its four cysteine ligands. The van der Waals parameters for the zinc ion were taken from AMBER ff99SB-ILDN force field [61]. The TIP3P force field [62] was used for the water molecules. All bond lengths were constrained by LINCS algorithm [63]. Periodic boundary conditions were applied. Electrostatic interactions were calculated using the Particle Mesh-Ewald (PME) method [64], and van der Waals and Coulomb interactions were truncated at 10 Å.

The systems underwent 1,000 steps of steepest-descent energy minimization with 1,000 kJ·mol^−1^·Å^−2^ harmonic position restraints on the protein, followed by 2,500 steps of steepest-descent and 2,500 steps of conjugate-gradient minimization without restraints. The systems were then gradually heated from 0 K up to 302 K in 20 steps of 2 ns. After that, equilibration was performed. Finally, 150 ns long productive MD simulations were carried out for each system in the NPT ensemble (constant temperature at 302 K, constant pressure at 1 bar and 2 fs time step by coupling the systems with a Nosé-Hoover thermostat [65,66] and an Andersen-Parrinello-Rahman barostat [67]). The most representative structure for each SIRT2/NAD^+^ model was identified by the cluster analysis [55] over the equilibrated trajectories, ranging from 30 ns to 120 ns.

#### Selection of the best full-length SIRT2/NAD^+^ model

In order to screen across the seven full-length SIRT2/NAD^+^ models, each of them was docked onto one of SIRT2's known cellular partners [15], the cyclin A/CDK2 complex [15]. Cyclin A/CDK2 (cCDK2) uses its cofactor ATP to phosphorylate serine or threonine residues of its substrates (the so-called ‘***P*** *’* site) followed by a proline, highly conserved across nearly all substrates (at the ***P+1*** site), and a basic residue at the ***P+3*** site [68–70]. Structural information has been provided by the complex between CDK2 and cyclin A bound to the HHASPRK peptide (the underlined residues are those recognized by the enzyme) [70].

Two models of cCDK2 were constructed (A and B hereafter). A is based on the X-ray structure of human CDK2 in complex with cyclin A and the HHASPRK peptide (PDB ID: 1QMZ [70]). This contains one Mg(II) ion bound to a water molecule, the ATP cofactor, and N132 and D145 of CDK2. B contains two Mg(II) ions and this species might be present *in vivo* [71]. The positions of the second Mg(II) ion and water molecules in model B was determined by superimposing the kinase domains of CDK2 (residues 5–209) with the ones of and protein kinase A (PKA) (PDB ID: 1ATP [72]) PKA (residues 44–245) using PyMOL (Molecular Graphics System, Version 1.3, Schrödinger LLC). The latter kinase shares 32% of sequence identity with CDK2, and its X-ray structure includes two metal ions and coordinated water molecules [72].

A and B were docked onto the HHASPRK peptide (used for protocol validation) and SIRT2. In the docking calculations, we considered only non-hydrogen atoms and imposed restraints between substrate's ***P*** site residue and ATP, because the contact between ATP and the ***P*** site residue is the prerequisite for the phosphoryl group transfer reaction [73], and between substrate's basic residue at the ***P+3*** site and CDK2's pT160 and cyclin A’s I270, because these contacts are crucial for substrate specificity, as indicated by the crystal structure of cCDK2•HHASPRK [70]. Distance restraints were applied on Mg(II) ions and their ligands to preserve the coordination structure using the HADDOCK program [74,75]. Rigid body docking, semi-flexible simulated annealing and water MD refinement were carried out.

In all docking calculations, adducts with A turned out to feature better HADDOCK scorings [74,75], number of best-scoring structures, and distance between ATP and the serine at the ***P*** site, than those of B (data not shown). Therefore the adducts with A are shown here. Our docking calculations further indicate that only one of the seven full-length SIRT2/NAD^+^ models could reproduce the conserved interaction patterns between cyclin A/CDK2 and its substrates as observed in crystal structure [70] (Model 7 in Fig 2). This model was used also to predict the structure of the full-length apo-SIRT2 model.

#### Modeling and MD simulations of full-length SIRT2 and SIRT2-pS331

The NAD^+^ cofactor was manually removed from the SIRT2/NAD^+^ model determined in previous steps, and then 150 ns long MD simulations were performed in a cuboid with edge lengths of 93 Å, 101 Å and 100 Å containing ~30,000 water molecules and five Na^+^ ions with the same computational set-up as described above. The most representative structure from the equilibrated trajectories (80–150 ns) was identified by the cluster analysis [55]. The most representative structure of SIRT2 was further used to predict the structure of the SIRT2-pS331 model. 500 ns long MD simulations were performed in a cuboid with edge lengths of 85 Å, 106 Å and 95 Å containing ~25,000 water molecules and seven Na^+^ ions with the same computational set-up as described above. The most representative structure from the equilibrated trajectories (200–500 ns) was identified by the cluster analysis [55].

#### Computational analysis

To assess the convergence of each simulated trajectory, we performed the Hess’s analysis [76]. Hydrogen bonds were defined to be present if the distance between the acceptor and donor atoms is below 3.5 Å and the angle among the hydrogen-donor-acceptor atoms are below 30 degree. Structural analysis was carried out using the tools implemented in GROMACS 4.5.5 code [55]. All figures for the visualization of structures were drawn using PyMOL (Molecular Graphics System, Version 1.3, Schrödinger LLC).

### Plasmids and recombinant proteins

Recombinant proteins were produced in E. coli BL21(DE)pLysS as GST fusion proteins using the constructs pGEX-SIRT2 (isoform 2) and pGEX-SIRT2-ΔCT (lacking the terminal 32 amino acids). Transformed bacteria were grown to an OD of 0.5–0.7 and protein expression was induced with 1 mM IPTG at 21°C for 16 h. Bacterial pellets were lysed in buffer containing 20 mM Tris-HCl, pH 8.0, 150 mM NaCl, 1 mM EDTA, 5 mM DTT, 1 mM Pefabloc SC, and 14 μg/ml Aprotinin, sonicated, and centrifuged at 10.000xg at 4°C for 30 min. GST-fusion proteins were separated from the cell lysate on glutathione-agarose (Sigma-Aldrich). The column was washed in 100 mM Tris-HCl pH 8.0 and 120 mM NaCl, and eluted in 100 mM Tris-HCl, pH 8.0, 120 mM NaCl, and 20 mM Glutathione. The purified proteins were analyzed on SDS-PAGE and Coomassie Blue staining.

### Deacetylation assays

For deacetylation assays 0.5 μg porcine tubulin (Cytoskeleton Inc.) was incubated in 50 mM Tris-HCl, pH 8.0, 10% (v/v) Glycerol, 1 mM DTT, 0.1 mM EDTA containing ProteoBlock protease inhibitor cocktail (Fermentas) with 1 μg of GST or GST fusion proteins. Tubulin (B-5-1-2, Abcam) and acetylated tubulin (K40ac antibody 6-11B-1, Sigma-Aldrich) were detected on Western Blots as described before [15]. Moreover we employed the SIRT2 activity assay kit (ab156066, Abcam, Cambridge, UK) according to the manufacturer’s protocol using GST-tagged fusion proteins (0.5 μg/assay).

## Results and Discussion

The two major SIRT2 isoforms 1 and 2 with 389 and 352 amino acids, respectively, can be divided into three parts, i.e. the catalytic core domain (CC) and N-terminal (NT) and C-terminal (CT) regions, which flank the CC (Fig 1A). The overall structure of the CC is conserved across all sirtuin members [18]. It contains a large, structurally homologous Rossmann fold (the so-called large domain [13]), a small, structurally diverse domain (the so-called small domain [13]) made up by a zinc binding pocket and a hydrophobic pocket, and four loops connecting the two domains (Fig 1B). In contrast the NT and CT show divergence in length and sequence among sirtuins [77,78]. They are disordered and are presumed to be highly flexible [18]. In the isoform 2 of SIRT2, modeled in this work, NT and CT are composed of amino acids 1–25 and 320–352, respectively.

In the following, we will first describe our models of full-length SIRT2 with and without NAD^+^ showing how the presence of the CT tail may affect the CC of the protein. Then we discuss the model of SIRT2 in the presence of pS331 modification. Specifically we show how phosphorylation of the CT might interfere with SIRT2 function by modifying the interaction of the CT with the NAD^+^ binding site.

### Models of full-length wild-type SIRT2

Full-length models of wild-type SIRT2 with and without NAD^+^ (SIRT2 and SIRT2/NAD^+^, respectively) were obtained by combining bioinformatics, modeling techniques and MD simulations. The stability, sampling and convergence of our MD simulations were established by calculating the backbone RMSD and by performing principal component (PC) analysis. The RMSD values (S1 and S2A Figs) for backbone atoms of the CC in SIRT2 and SIRT2/NAD^+^ oscillate around 2 Å compared to the X-ray structure [13]. The NT and CT regions, as expected, have much larger RMSD. The RMSD values of NT and CT reach a plateau after around 130 ns and 80 ns in the SIRT2/NAD^+^ and SIRT2 MD simulations, respectively (S1 and S2A Figs). The sampling and convergence of our MD simulations were further checked by the so-called Hess analysis [76]. In this analysis, the trajectories’ projections on the top essential dynamical spaces obtained from a standard covariance analysis are considered. These projections are next compared with those expected for a random reference. The observed negligible overlap (i.e. cosine content close to 0) that we found for our systems (cosine contents was 0.022, 0.002 and 0.131 for the first three PCs in SIRT2/NAD^+^, and 0.037, 0.036, and 0.116 for the first three PCs in SIRT2) confirms a posteriori an adequate sampling of our systems around the equilibrium position. In the following, we will describe the structural features of our models for each domain.

The modeled structures show a conserved CC domain highly similar to the X-ray structure [13] (RMSD values are 2.2±0.2 Å and 2.6±0.2 Å for SIRT2/NAD^+^ and SIRT2, respectively), a structurally variable and partially disordered NT, while the presence of a short helical region is predicted for CT (Fig 1C).

#### The NT region

The NT extension is located below the large domain of CC in both the SIRT2/NAD^+^ and the SIRT2 models, far away from the active site (Fig 1C). This suggests that the NT does neither influence NAD^+^ nor substrate binding directly. This is consistent with the observation that the NT has no effect on the *in vitro* deacetylase activity of SIRT2 [13]. However, indirect effects on SIRT2 activity cannot be excluded, e.g. by modulating protein-protein interactions involved in substrate recognition and in subcellular targeting of the enzyme.

#### The CC domain

**Fig 3:**
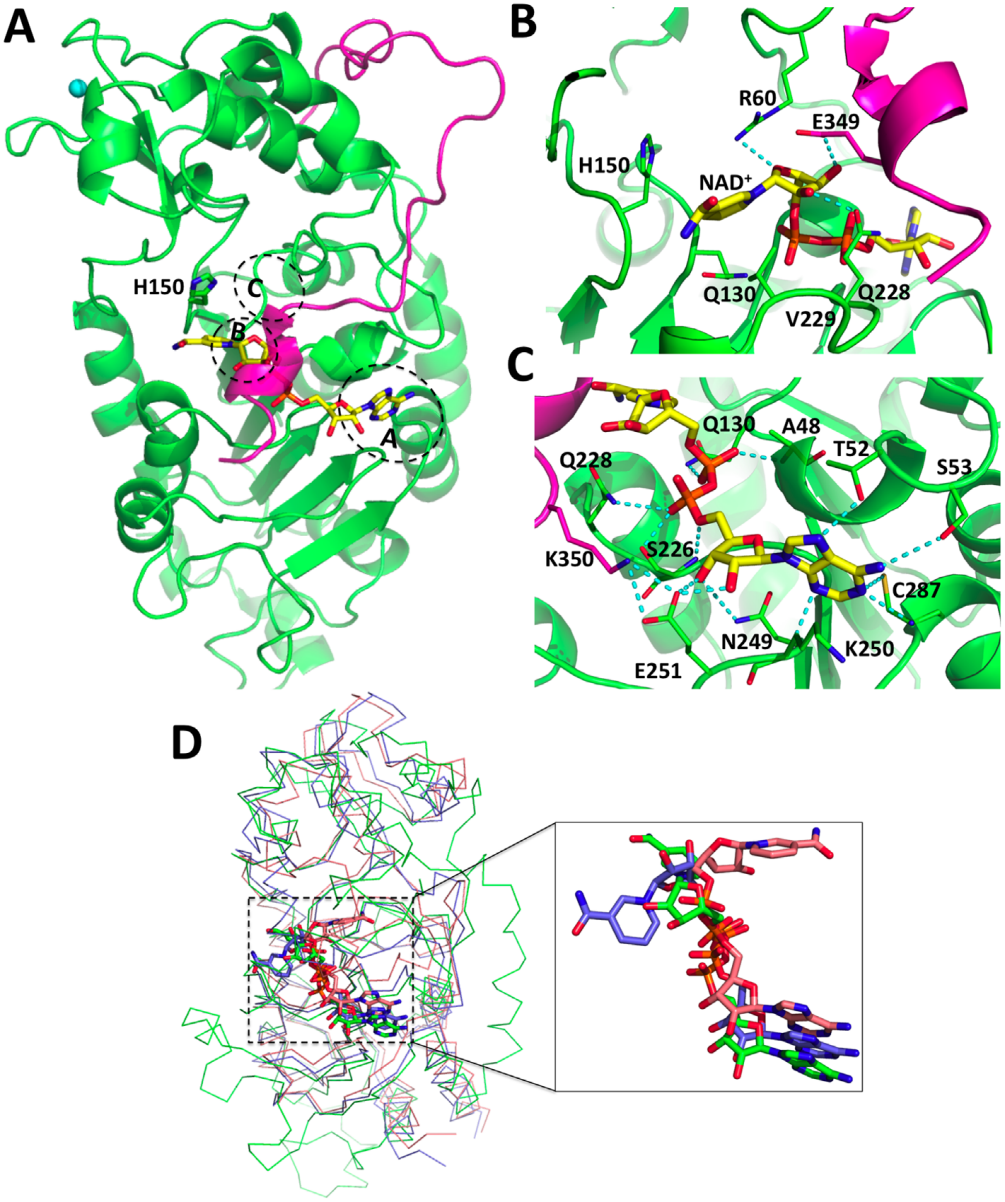
The NAD^+^ binding site in full-length SIRT2. **(A)** Overview of the SIRT2/NAD^+^ complex. The NAD^+^ (yellow) and catalytic residue H150 (green) are represented by sticks. The CT is highlighted in pink. The sites A, B and C of SIRT2, which are involved in NAD^+^ binding, are indicated by blue circles. **(B)** Close-up view of the binding site of nicotinamide and nicotinamide ribose. **(C)** Close-up view of the binding site of adenine. Hydrogen bonds are highlighted by dashed lines. **(D)** Superposition of two crystal structures of Sir2-Af2/NAD^+^ (PDB ID: 1S7G [16]) with the SIRT2/NAD^+^ complex (in green). The chain A (NAD^+^ in non-productive conformation) and chain B (NAD^+^ in productive conformation) of 1S7G are colored in blue and salmon, respectively. The NAD^+^ cofactors are represented by sticks.

Some of the reported X-ray structures of CC domain of sirtuins host the NAD^+^ cofactor required for enzymatic activity [14,16–19]. The NAD^+^ binding pocket is located across the Rossmann fold and the hydrophobic pocket (Fig 3A–3C). The NAD^+^ binding pocket can be divided into three regions (A, B and C sites) [14]: site A, where the adenine ribose moiety of NAD^+^ is bound; site B, where the nicotinamide ribose of NAD^+^ is bound; and site C, where the nicotinamide moiety of NAD^+^ is bound. The location of nicotinamide characterizes a productive or a non-productive state for activating NAD^+^ [16–18] (Fig 3D). In the former, the nicotinamide ribose is hydrogen bonded with the acetyl-lysine carbonyl oxygen of the substrate, which would allow the nucleophilic attack on the nicotinamide ribose for the deacetylation catalysis [16–18]. In the latter NAD^+^ is not properly positioned in site C (Fig 3A) [14,16,19]. As a result, the nicotinamide moiety of NAD^+^ fails to interact with key residues required for catalysis and the complex is not productive.

**Fig 4:**
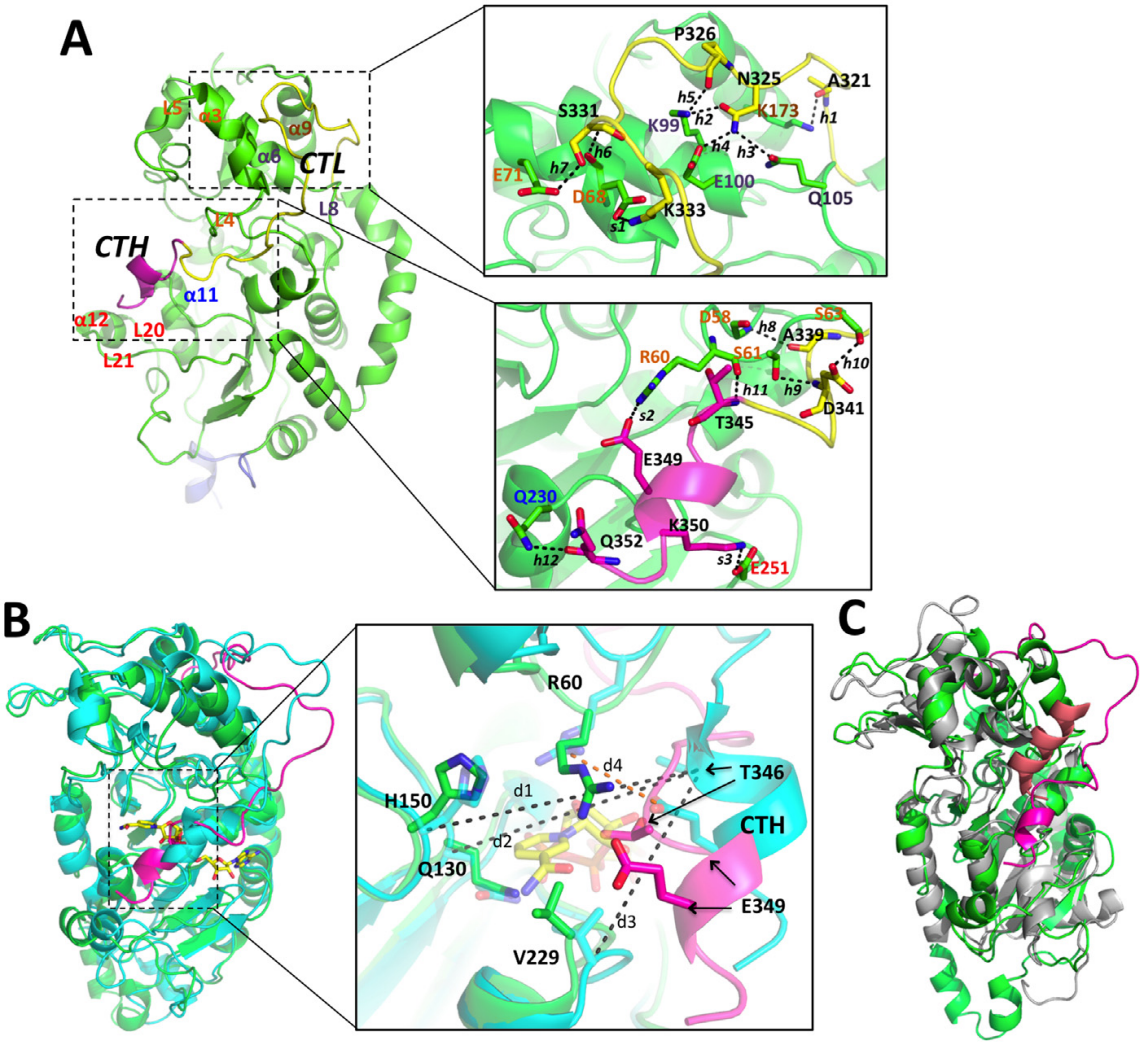
Interaction of the CT region with the NAD^+^ binding site. **(A)** Detailed view of the interactions between the CC and the CT in the SIRT2 model. The CC, NT, CTL and CTH are colored in green, blue, yellow and pink, respectively. CTL and CTH refer to the C-terminal loop and the C-terminal α-helix, respectively, of the CT. The hydrogen bonds are represented by dashed lines. The details of the labeled hydrogen bonds and salt bridges are shown in Table 3. **(B)** Superposition of the most representative configurations of SIRT2/NAD^+^ (cyan) and SIRT2 (green). The CT of SIRT2 model is colored in pink. The NAD^+^ cofactor in the SIRT2/NAD^+^ is represented by yellow sticks. See Table 4 for the definition of the measured atomic distances (d1, d2, d3 and d4). The two arrows from T346 and E349 refer to the two positions of these amino acids in the SIRT2 and the SIRT2/NAD^+^ models. **(C)** Superposition of the most representative configuration of SIRT2 (green) with the crystal structure of yeast Hst2 [79] (gray). The CT regions of SIRT2 and Hst2 are colored in red and salmon, respectively.

Both full-length proteins with and without NAD^+^ are, not unexpectedly, architecturally similar to the previously reported CC crystal structure (Fig 1C). However, the insertion loop consisting of *L21-α12-L22* (residues 250–280) along with the *α11* helix (residues 230–236), and the cofactor binding loop *L4-α3-L5* (residues 55–80, one of the most conserved regions of sirtuins [79–82]), rearrange considerably in the presence of the CT because of multiple interactions (Fig 4A). This is of special importance, as they may play a role in NAD^+^ binding and substrate recognition [13]. In particular, the insertion loop has been suggested to be important for substrate selection [13,83]. Thus the CT is likely to contribute, indirectly, to NAD^+^ binding and substrate specificity.

**Table 3:** Hydrogen bond and salt bridge interactions between the CC and the CT in SIRT2.

Interaction ID	Residue ^a^	Residue ^b^	Distance (Å) ^c^
h1	A321	K173	3.0 (0.3)
h2	N325	K99	2.9 (0.3)
h3	N325	Q105	3.0 (0.2)
h4	N325	E100	3.6 (0.9)
h5	P326	K99	2.9 (0.2)
h6	S331	D68	3.8 (1.0)
h7	S331	E71	4.4 (1.9)
h8	A339	D58	3.0 (0.2)
h9	D341	S61	3.1 (0.3)
h10	D341	S63	3.4 (0.7)
h11	T345	R60	3.6 (1.0)
h12	Q352	Q230	3.3 (0.8)
s1	K333	D68	3.5 (1.1)
s2	E349	R60	3.9 (1.9)
s3	K350	E251	4.5 (1.4)

Standard deviations are given in parentheses.

^*a*^ Residues belong to the CT.

^*b*^ Residues belong to the CC.

^*c*^ Atomic distances (h1–h12) between donor and acceptor, and center-of-mass distances (s1–s3) between negative and positive side chains are calculated on the equilibrium trajectory from MD simulations of SIRT2.

In our SIRT2/NAD^+^ model, NAD^+^ is bound in a non-productive state (Fig 3A). It binds to a large groove between the Rossmann fold and the hydrophobic pocket of the smaller domain, similar to the previously reported structures of sirtuins/NAD^+^ [14,16–19]. In this conformation the nicotinamide moiety of NAD^+^ forms hydrophobic contacts with Q130, H150 and V229 around the active site (Fig 3A). Such weak interactions between nicotinamide and SIRT2 imply a larger conformational flexibility of nicotinamide in the substrate-free SIRT2, consistent with the variability of the nicotinamide binding position observed in previously solved crystal structures [14,16,19]. Such flexibility may ease the passing of NAD^+^ from the non-productive to the productive state, accompanied with the rotation of the nicotinamide (shift from hydrophobic pocket to interacting with site C) and nicotinamide ribose (bound to site B) of NAD^+^ in the presence of substrates [84]. The nicotinamide ribose in site B forms two hydrogen bonds with CC residues, R60 with the oxygen of the ribose ring and Q228 with the hydroxyl group at position 2′′, and a hydrogen bond between E349 of the CT and the hydroxyl group at position 3′′ (Fig 3B). The adenine base (bound to site A) sits in a partially hydrophobic pocket. It forms several hydrogen bonds with the side chains of T52, S53, C287 and the highly conserved N249 (93% of conservation across all sirtuin members in all species [18]), and with K250 and C287 backbone atoms (Fig 3C). E251, conserved between SIRT1, SIRT2, SIRT5, Sir2-Af2 from *A*. *fulgidus* and yeast Hst2 (a protein homolog to SIRT2 with sequence identity of 42%), makes hydrogen bonds with both OH groups of the adenine ribose, and is salt bridged with K350 of the CT. The oxygen atoms of the phosphate groups that connect the two ribose groups make several hydrogen bonds with Q130, S226 and Q228 (Fig 3C).

#### The CT region

The CT in both our predicted wild-type full-length models, i.e. with or without NAD^+^ (Fig 1C), contains a short helix (CTH hereafter, residues T345-K350) and a longer disordered loop (CTL hereafter, residues G320-R344) connecting the CTH with the CC (Fig 4A). CTL adapts a turn conformation around the *L4-α3* of the CC, making extensive interactions with the cofactor binding loop and the *α9* of the zinc-binding domain (Fig 4A). CTH binds at the edge of the nicotinamide ribose-binding site (site B) between the large Rossmann fold and the hydrophobic pocket in the small domain (Fig 4A) in both SIRT2 and SIRT2/NAD^+^. However, CTH is locked deeper into the active site of SIRT2 than SIRT2/NAD^+^, because T346 in CTH occupies the binding site of the hydroxyl groups of NAD^+^ nicotinamide ribose (Fig 4B). In particular, the Cα distances between T346 and H150, Q130, and V229 of the CC decrease about 2 Å (Table 4) in SIRT2 with respect to SIRT2/NAD^+^. Furthermore CTH residue E349 forms a salt bridge with R60 in the cofactor-binding loop. This stabilizes the interaction of the CTH with the active center in the absence of NAD^+^. Therefore, the SIRT2 CT may function as an autoinhibitory region to regulate the deacetylation activity by partially occluding the binding site of NAD^+^, and therefore decreasing its binding affinity for NAD^+^.

**Table 4:** Average atomic distances (in Å) between Cα of T346 and that of H150, Q130 and Q229, and between Cς of R60 and C of E349.

	Atomic distances	SIRT2/NAD^+^	SIRT2	SIRT2-pS331
**d1**	Cα of H150—Cα of T346	15.5 (1.9)	13.5 (0.8)	12.3 (0.9)
**d2**	Cα of Q130—Cα of T346	14.2 (1.8)	12.1 (0.8)	11.0 (1.1)
**d3**	Cα of V229—Cα of T346	11.9 (1.0)	8.6 (0.9)	6.6 (0.8)
**d4**	Cς of R60—Cδ of E349	7.1 (2.3)	4.5 (1.5)	13.4 (4.6)

Standard deviations are given in parentheses.

In SIRT2/NAD^+^ the E349 of the CT forms hydrogen bond interactions with the nicotinamide ribose. This may increase the energy barrier of NAD^+^ for passing from the non-productive to the active productive state, which is unfavorable for trigging the following deacetylation reaction.

**Fig 5:**
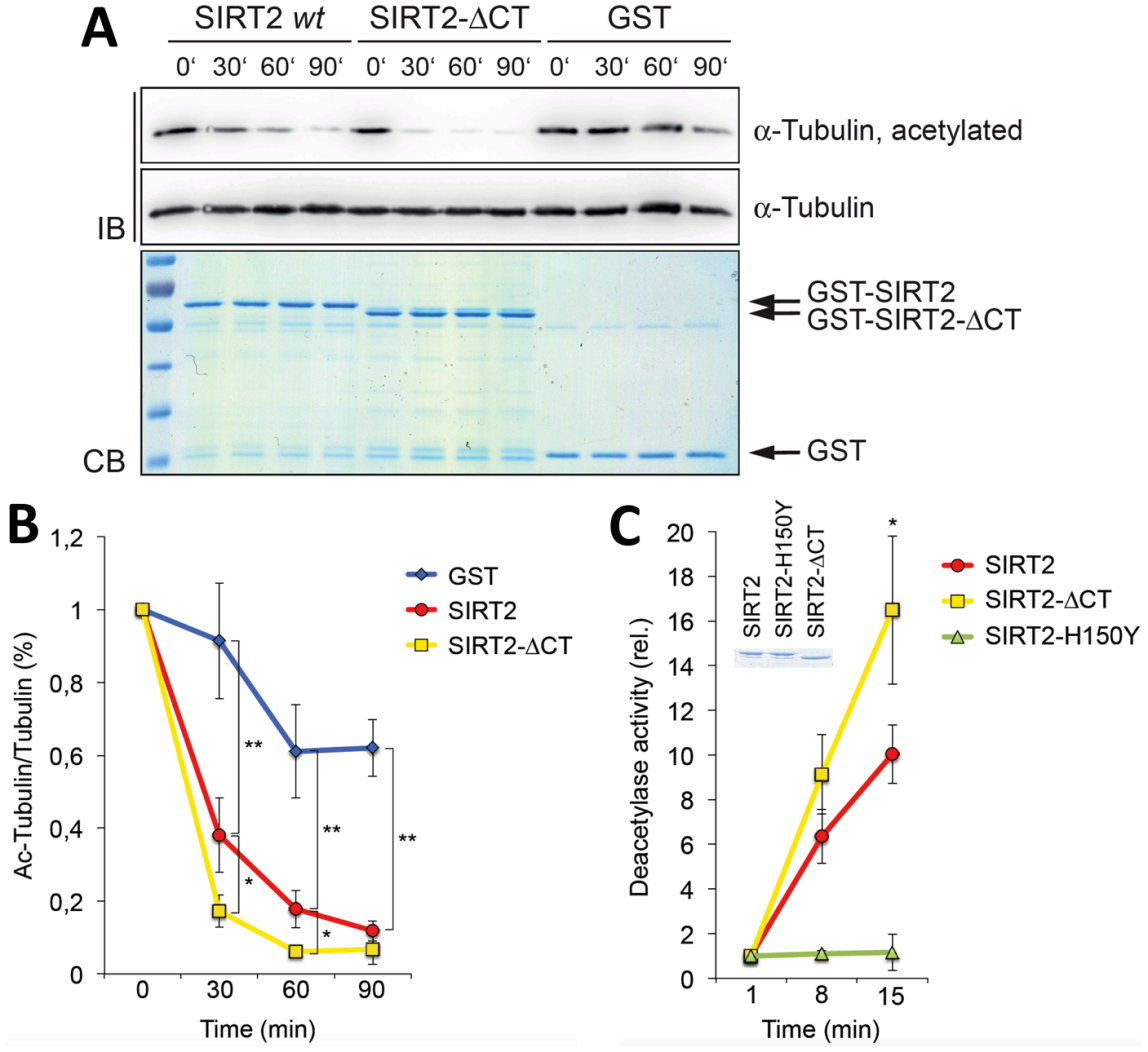
Tubulin deacetylation assay with SIRT2. **(A)** GST-SIRT2 wild-type (wt) or a C-terminally truncated GST-SIRT2-ΔCT fusion proteins were incubated with commercially available purified tubulin in the presence of NAD^+^ for the indicated periods of time (0 to 90 minutes). GST was included as a negative control. The samples were subjected to SDS-PAGE and either stained by Coomassie blue (CB) or tubulin and acetylated tubulin were measured by Western blot analysis using specific antibodies (IB). **(B)** Quantification of three independent experiments. Mean values and standard deviations are indicated. **(C)** GST-SIRT2 wild-type, catalytically inactive GST-SIRT2-H150Y or GST-SIRT2-ΔCT fusion proteins were assayed using a fluorometric SIRT2 substrate. Three independent experiments were quantified, mean values and standard deviations are indicated. The insert shows the GST fusion proteins used in the assays. Statistical significance was evaluated using a two-sided students T-test: * p<0.05, ** p<0.01.

We tested the hypothesis that the CT of SIRT2 has an autoinhibitory function by measuring the catalytic activity of full-length SIRT2 and of SIRT2 with a deletion of the CT using GST fusion proteins. We incubated GST-SIRT2, C-terminally truncated GST-SIRT2-ΔCT, or GST alone for control with commercially available purified tubulin in the presence of NAD^+^. In time course experiments we observed that SIRT2-ΔCT was more efficient in deacetylating tubulin at K40 compared to the wild-type protein (Fig 5A and 5B) and in deacetylating a SIRT2 selective fluorogenic substrate peptide (Fig 5C). A catalytically inactive mutant of SIRT2, GST-SIRT2-H150Y, did not show activity towards the peptide (Fig 5C). These findings are consistent with the postulated activity of the CT in partially occupying the NAD^+^ binding pocket.

An autoinhibitory role of CT domains has also been observed *in vitro* for other sirtuins, i.e. SIRT3 [85] (a member of the same sirtuin sub-class as SIRT2 [18]) and Hst2 [79]. In the latter the CT (which is much longer, i.e. 62 amino acids instead of 33 in SIRT2, see Fig 1A) completely occludes the active site [79] (Fig 4C). Thus our models suggest that modifications of the CT (e.g. by phosphorylation and acetylation) might affect sirtuin deacetylase activities by changing the location and folding of CTH and thereby regulating the loading and positioning of NAD^+^ in the active center. Therefore we suggest that alterations in the flexibility of the CT or part of the CT due to post-translational modifications may modify its autoinhibitory activity by either enhancing or diminishing the interaction of the CT with the NAD^+^ binding pocket. In turn this might then affect the binding or the positioning of the NAD^+^, which are important determinants of catalytic activity.

### SIRT2-pS331

**Fig 6:**
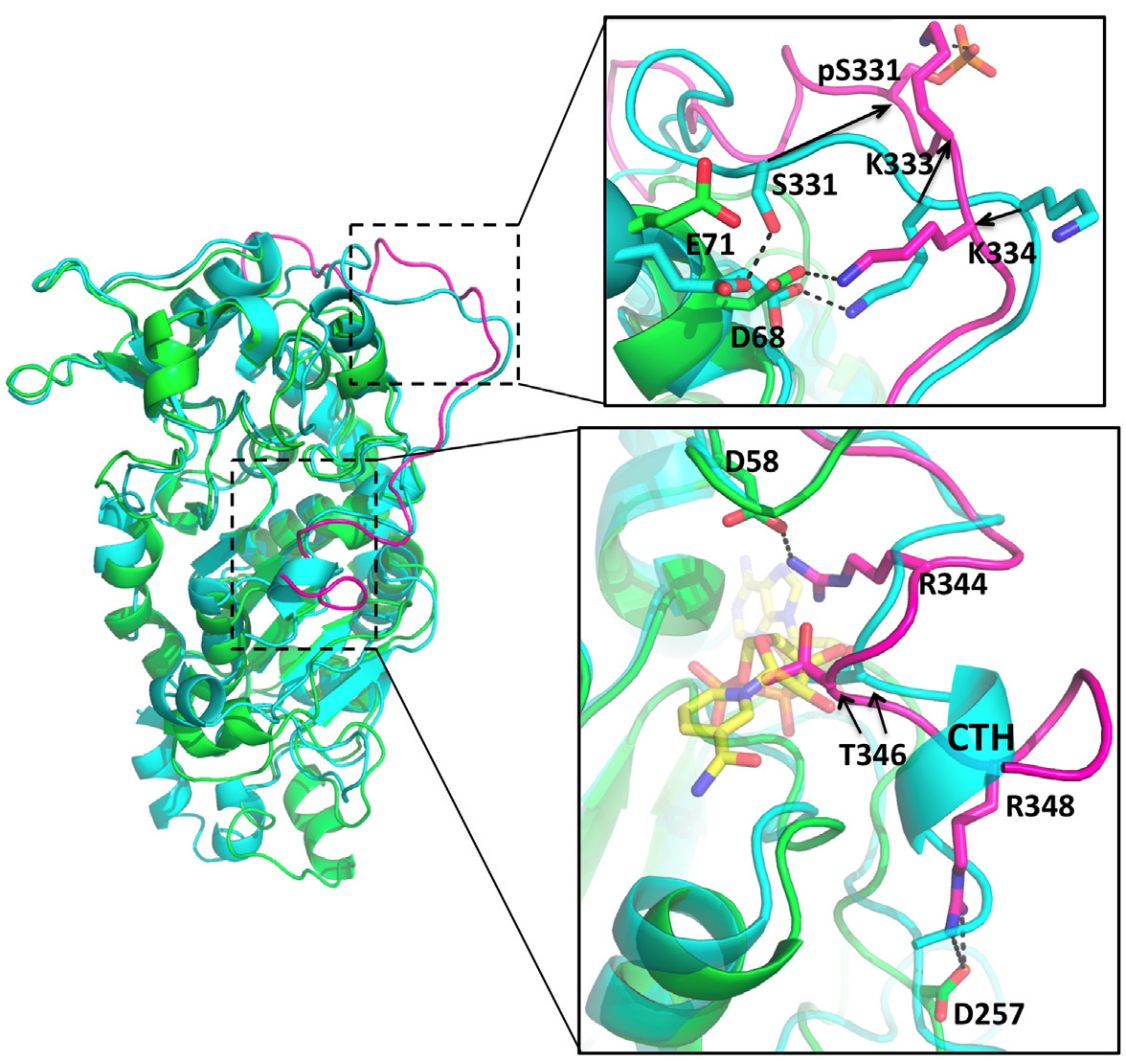
Interaction of the S331 phosphorylated CT region with the NAD^+^ binding site. Superposition of the most representative configurations of SIRT2 (cyan) and SIRT2-pS331 (green). The CT of SIRT2-pS331 is colored in pink. The NAD^+^ cofactor in the SIRT2/NAD^+^ is represented by yellow sticks.

Our previous study has provided evidence that S331 phosphorylation interferes with the catalytic activity of SIRT2 both in vitro and in cells [15]. As S331 is located in the CT, our findings are consistent with the suggestion that the CT affects the deacetylation activity and that this is modulated by phosphorylation. To evaluate the hypothesis that phosphorylation of the CT at S331 interferes with SIRT2 function by altering the interaction of the CT with the CC, we modeled SIRT2 with phosphorylation at S331 (SIRT2-pS331). The good sampling and convergence of the 500 ns long MD simulations on SIRT2-pS331 were confirmed by the PC analysis, i.e. the cosine contents for the first three PCs are 0.320, 0.231, and 0.369. While the CC domain does not change significantly (backbone RMSD values are 2.4±0.2 Å with respect to the X-ray structure [13], see S2B Fig), the CT shows large conformational changes with its average backbone RMSD (S2B Fig) during the simulations being 4.9±0.7 Å (Fig 6). Due to the repulsion between pS331 and E71 and D68 in the cofactor binding loop, the backbone of the phosphorylated CT moves ~10.7 Å away from the CC. pS331 forms a salt bridge with K333, and D68 salt bridges with K334. As a result, the CTH becomes fully unfolded and the autoinhibitory region moves deeper into the NAD^+^ binding pocket by ~1.5 Å compared to the unphosphorylated SIRT2 (Table 4 and S3 Fig). This new configuration of the CTH is stabilized by two salt bridges formed between R344 in the CT and D58 in the CC, and between R348 in the CT and D257 in the CC. T346 occupies the presumed binding site of nicotinamide ribose (Fig 6). The results of our simulations support the hypothesis that phosphorylation of the CT at amino acid S331 induces a conformational change that contributes to the regulation of the autoinhibitory region of the CT. These findings are consistent with the previous biochemical findings that phosphorylation of S331 inhibits SIRT2 catalytic activity [15]. In broader terms other modifications of the CT may also affect the interaction of the CT with the NAD^+^ binding site and thus regulate catalytic activity of SIRT2.

## Conclusions

In summary, we present full-length molecular models of apo-SIRT2, the SIRT2/NAD^+^ complex and SIRT2-pS331 by using bioinformatics tools and MD simulations. Based on the models and the functional analysis of the CT and of the phosphorylation at S331, we suggest that the C-terminal region of SIRT2 functions as an autoinhibitory region that regulates the deacetylation activity of SIRT2. This can occur by increasing the stability of the non-productive NAD^+^ in SIRT2/NAD^+^ and/or partially occupying the NAD^+^ binding site in apo-SIRT2. The inhibitory role of the CT region is consistent with the increased catalytic activity of a SIRT2 mutant that lacks this region. Phosphorylation at S331 causes large conformational changes in the CT that enhance the autoinhibitory activity of the CT region. This is consistent with our previous findings that CDK-dependent modification of S331 inhibits SIRT2 catalytic activity both *in vitro* and in cells [15]. Together these findings suggest that the CT region of SIRT2 might function more generally in integrating signals that target and regulate this deacetylase during cellular processes. Moreover the molecular insight into the role of the CT region in controlling SIRT2 function provided by our work might help to design specific inhibitors to control SIRT2 deacetylation activity for therapeutic applications.

## Supporting Information

S1 Fig Backbone RMSD of the entire SIRT2 (black line), CC (red line), CT (green line), NT (blue line), and NAD^+^ (purple line) is plotted as a function of the simulated time for SIRT2/NAD^+^. The respective starting conformations are considered as reference structures.
(DOCX)

S2 Fig Backbone RMSD of the entire protein (black line), CC (green line), CT (red line) and NT (blue line) is plotted as a function of the simulated time for SIRT2 (A) and SIRT2-pS331 (B). The respective starting conformations are considered as reference structures.
(DOCX)

S3 Fig The centers-of-mass distance between the Cα atoms of CTH residues (T345-K350) and those of the NAD^+^ binding site in CC (H150, Q130, and V229) is plotted as a function of simulated time for SIRT2-pS331 system. (DOCX)
