# Supplementary material for: Insight into the Mechanism of Intramolecular Inhibition of the Catalytic Activity of Sirtuin 2 (SIRT2)
Source: PLoS One. 2015 Sep 25;10(9):e0139095. doi: 10.1371/journal.pone.0139095 (PMC4583397; doi:10.1371/journal.pone.0139095)
Supplement: S1 Fig — The respective starting conformations are considered as reference structures. (DOCX) [file pone.0139095.s001.docx]

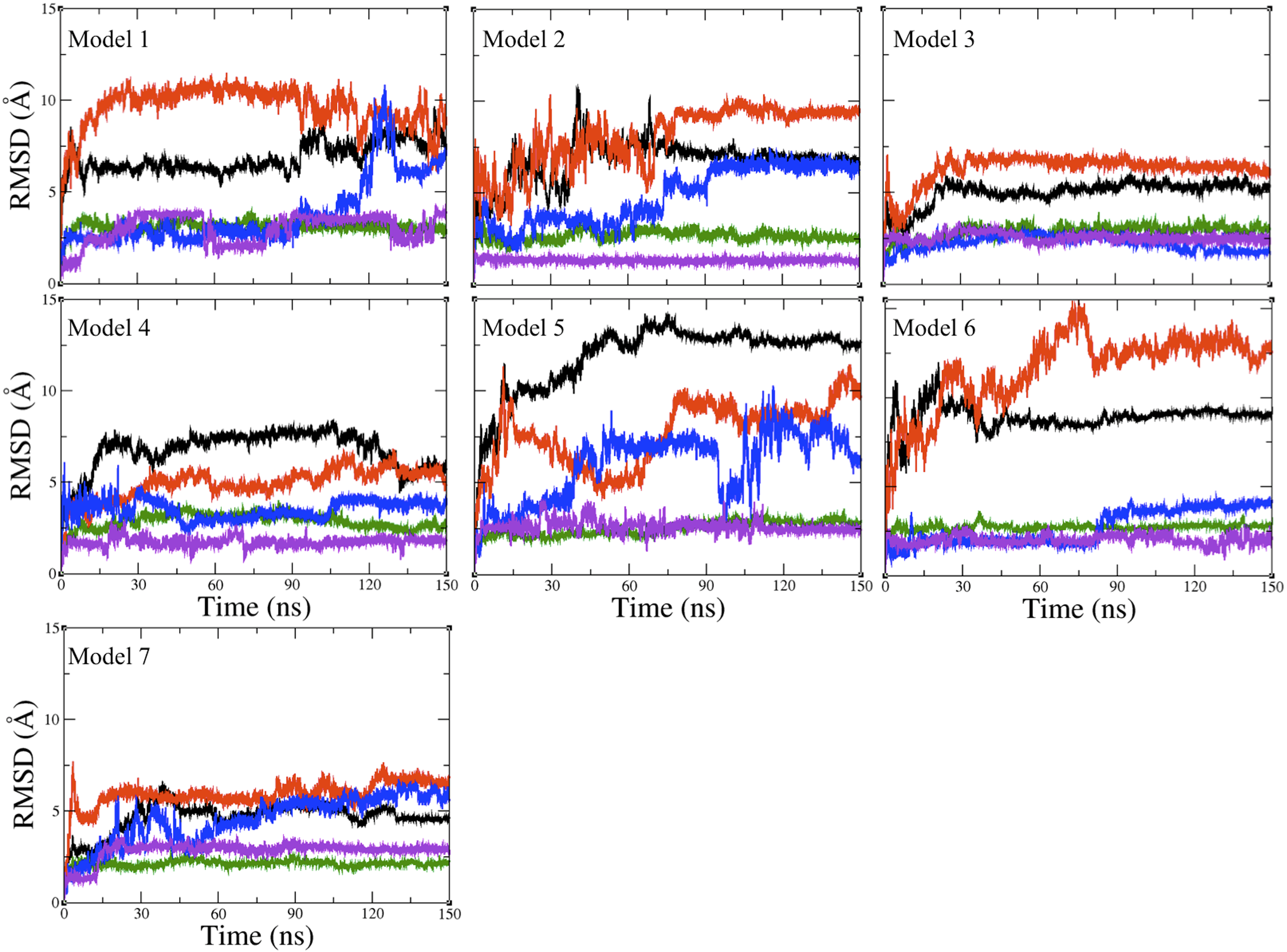


**S1 Fig. Backbone RMSD of the entire SIRT2 (black line), CC (red line), CT (green line), NT (blue line), and NAD^+^ (purple line) is plotted as a function of the simulated time for SIRT2/NAD^+^. The respective starting conformations are considered as reference structures.**
