# Supplementary material for: Insight into the Mechanism of Intramolecular Inhibition of the Catalytic Activity of Sirtuin 2 (SIRT2)
Source: PLoS One. 2015 Sep 25;10(9):e0139095. doi: 10.1371/journal.pone.0139095 (PMC4583397; doi:10.1371/journal.pone.0139095)
Supplement: S2 Fig — The respective starting conformations are considered as reference structures. (DOCX) [file pone.0139095.s002.docx]

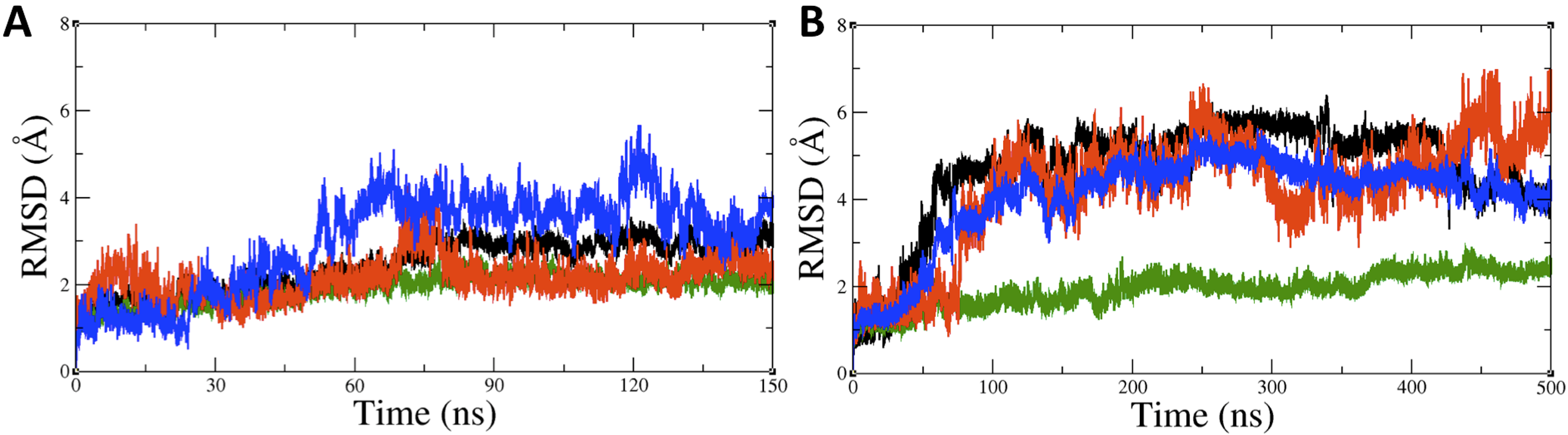


**S2 Fig. Backbone RMSD of the entire protein (black line), CC (green line), CT (red line) and NT (blue line) is plotted as a function of the simulated time for SIRT2 (A) and SIRT2-pS331 (B). The respective starting conformations are considered as reference structures.**
