# Supplementary material for: Insight into the Mechanism of Intramolecular Inhibition of the Catalytic Activity of Sirtuin 2 (SIRT2)
Source: PLoS One. 2015 Sep 25;10(9):e0139095. doi: 10.1371/journal.pone.0139095 (PMC4583397; doi:10.1371/journal.pone.0139095)
Supplement: S3 Fig — (DOCX) [file pone.0139095.s003.docx]

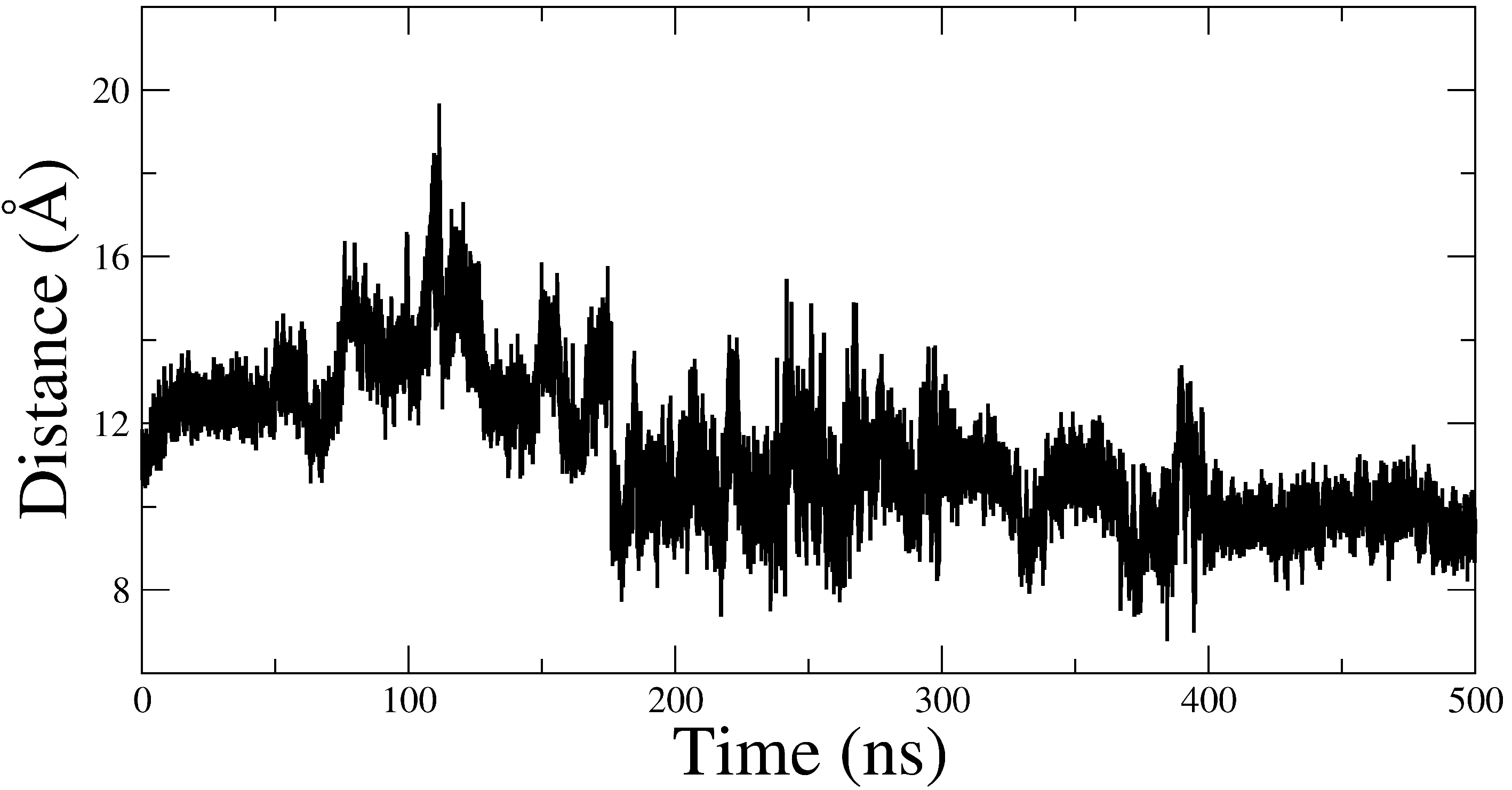


**S3 Fig. The centers-of-mass distance between the Cα atoms of CTH residues (T345-K350) and those of the NAD^+^ binding site in CC (H150, Q130, and V229) is plotted as a function of simulated time for SIRT2-pS331 system.**
